# Time Trends in Fast Food Consumption and Its Association with Obesity among Children in China

**DOI:** 10.1371/journal.pone.0151141

**Published:** 2016-03-14

**Authors:** Hong Xue, Yang Wu, Xiaoyu Wang, Youfa Wang

**Affiliations:** 1 Systems-oriented Global Childhood Obesity Intervention Program, Department of Epidemiology and Environmental Health, University at Buffalo, State University of New York, Buffalo, New York, United States of America; 2 Department of Health, Behavior and Society, Bloomberg School of Public Health, Johns Hopkins University, Baltimore, Maryland, United States of America; 3 Department of Health Policy and Management, Bloomberg School of Public Health, Johns Hopkins University, Baltimore, Maryland, United States of America; Stony Brook University, Graduate Program in Public Health, UNITED STATES

## Abstract

**Objective:**

Study the trends in Western fast food consumption (FFC) among Chinese school-age children and the association between FFC and obesity using nationwide survey data.

**Design:**

Cross-sectional and longitudinal analyses were conducted to study the trends in FFC and the associations between FFC and weight status (overweight, obesity and body mass index (BMI) z-score).

**Setting:**

Longitudinal data from families were collected in the 2004 and 2009 China Health and Nutrition Survey (covering nine provinces throughout China).

**Subjects:**

The analysis included 2656 Chinese children aged 6 to 18 years (1542 and 1114 children in the 2004 and 2009 survey, respectively).

**Results:**

FFC (reported having consumed Western fast food in the past three months) has increased between 2004 and 2009, from 18.5% to 23.9% in those aged 6–18, and increased more rapidly among those aged 13–17, from 17.9% to 26.3%. The increase was significant in almost all groups by age, sex, family income, and residence. Our cross-sectional and longitudinal analyses did not detect a significant association between FFC and obesity/overweight or BMI z-score (e.g., for BMI z-score, boys: β = 0.02, 95% CI: -0.71, 0.75; girls: β = -0.14, 95% CI: -1.03, 0.75).

**Conclusions:**

FFC has increased in Chinese school-age children, especially in older children, boys, and those from low- and medium-income families, rural areas, and East China, but decreased among those from high-income families during 2004–2009. The data did not show a significant association between FFC and obesity.

## Introduction

The recent three decades saw a growing global obesity epidemic; and overweight and obesity rates among children have increased in many countries including China[1–3]. In the U.S., the prevalence of childhood overweight and obesity has tripled since the 1970s[4]. In China, overweight and obesity prevalence has increased rapidly in children, from less than 3% overall in 1985 to approximately 10% in girls and 20% in boys in 2010, and the overall rate in major cities like Beijing is over 20%[3,5].

There has been a strong interest in developing effective childhood obesity prevention programs[6]. Along with the increasing obesity prevalence, research suggests that rapid economic development, urbanization, globalization, and changes in government policies followed by China’s entry of World Trade Organization (WTO) have led to emerging Western fast food outlets and dietary shifts among the Chinese population[7,8], which may have fueled the growing obesity epidemic. For example, since the first American fast food chain, Kentucky Fried Chicken (KFC), opened its first restaurant in China in 1987, the number of KFC restaurants has increased to over 4,200 in more than 800 cities and towns by 2012[9]. McDonald’s added 200 restaurant in 2011, and grew to over 2,000 restaurants within three decades in China[10]. In 2002, the fast food industry yielded 200 billion Chinese Yuan annual sales (approximately US$24 billion), accounting for 2/5 of China’s food and beverage sales[11].

The relationship between Western fast food consumption (FFC) and weight status remains mixed in the existing literature. Some research suggests a positive association while others do not [5,12]. Moreover, only a few cohort studies have tested the influence of FFC on obesity [13,14]. Very limited longitudinal studies have been conducted to examine the effect of FFC on weight status in children, and no study has been conducted in Chinese children[12].

This study examined the changes over time in FFC and tested the association between FFC and obesity (including overweight) among children in China using nationwide longitudinal data. We hypothesized that FFC had increased over time in China and FFC increased obesity risk in children.

## Materials and Methods

### Study design

**Fig 1:**
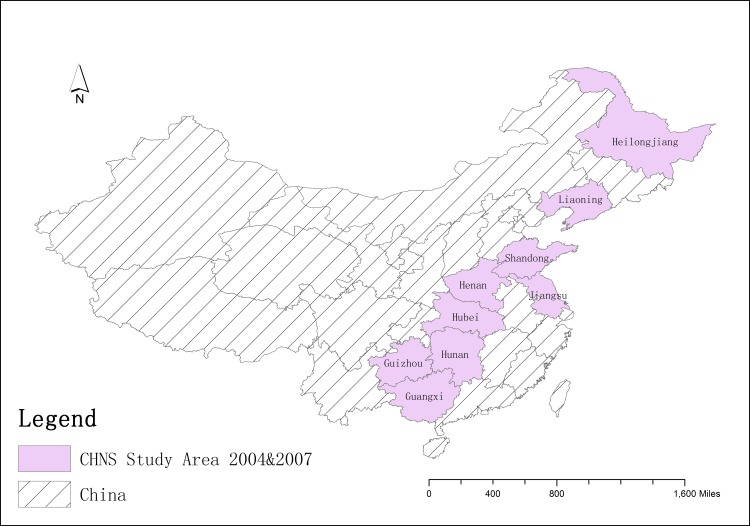
Map of the China Health and Nutrition Survey (CHNS) coverage 2004–2009.

We conducted both cross-sectional and longitudinal analyses using longitudinal survey data collected in the 2004 and 2009 China Health and Nutrition Survey (CHNS), which covered nine provinces throughout China[15]. **Fig 1**shows the map of the CHNS coverage for these waves.

CHNS is a large-scale, household-based open cohort which includes about 4,400 households and 26,000 individuals in the nine provinces, namely Heilongjiang, Liaoning, Shandong, Henan, Jiangsu, Hubei, Hunan, Guizhou, and Guangxi. CHNS used a multi-stage, random cluster sampling scheme to collect nationally representative data that covered key public health risk factors and health outcomes, demographic, social and economic factors at the individual, household and community levels[16]. At the individual level, detailed health-related data were measured, including dietary intake, physical activity, smoking and drinking behaviors, anthropometrics, blood pressure and limited clinical data[16].

### Study Sample

Our study sample was from the 2004 and 2009 CHNS because FFC data were not available before 2004. The question on FFC was only asked among those aged 6 years or older. Thus, we included children aged 6 to 18. Observations with missing body mass index (BMI) information were removed. A total of 293 observations were missing for BMI. By removing the observations with missing BMI, we obtained a final sample of 2656 observations, with 1542 children in 2004 and 1114 children in 2009, and among them, 376 individuals had complete data in both waves. Children included in the analytic sample were similar to those in the non-analytic sample except they were younger, had lower in-school physical activity levels and were less sedentary.

### Key Study Variables and Data Collection

BMI and obesity status: BMI was calculated as measured weight in kilograms divided by height in meters-squared. During the survey, weight and height data were collected by trained health workers from individual’s comprehensive physical exam at the local clinic or at the respondent’s home during each visit[17]. Height was measured without shoes to the nearest 0.2 cm using a portable stadiometer, and weight was measured without shoes and in light clothing to the nearest 0.1 kg on a calibrated beam scale (Seca North America, Chino, CA, USA).

Overweight and obesity in children were defined based on the International Obesity Task Force (IOTF)’s gender- and age-specific BMI cut-off points for children aged 2–18 years of old, which correspond to an adult BMI of 25 kg/m^2^ (overweight) or 30 kg/m^2^ (obesity) [18]. Children’s BMI varies with age and gender; thus, we calculated BMI z-scores adjusting for age and sex.

#### FFC

WesternFFC, excluding Chinese fast food. This definition is used throughout the entire article. Data for FFC were obtained from self-reports on this question in CHNS, “During the past 3 months, how many times have you eaten at a Western fast food restaurant, such as McDonald’s or Kentucky Fried Chicken?” Responses to this question were dichotomized into 1: consumed at least once or 0: not consumed or unknown to calculate the percentage of fast food consumers. Note that there were few FF restaurants (13.32%) in rural China and children were unlikely to eat in places outside 30 minutes’ bus ride from where they lived; therefore, we conducted imputation and treated the missing responses and those answered "unknown" as “0” if children lived in communities with no access to FF restaurants, which saved 2.28% of the final sample.

### Other Covariates

#### Urbanicity

Urban or rural residents depending on the administrative definitions of the communities in which the participants lived.

#### Ethnicity

Ethnic groups were dichotomized into Han or not, as Han is the majority ethnic group in China, while some minority groups share different habits and norms.

#### Family income

Income groups were categorized based on annual per capita household income tertiles in that wave. Annual per capita household income was adjusted to 2011 yuan currency values.

#### Physical activity

We looked at specific metabolic equivalent time (MET) intensity values, including in-school and before or after-school physical activity, transportation to/from school, and sedentary activities, which were retrieved from children’s self- reports. Based on the Compendium of Physical Activities, one unit of MET is defined as the ratio of a person’s working metabolic rate relative to his/her resting (basal) metabolic rate, and the final unit for physical activity variables is MET in kcal/(kg*h)[19]. We aggregated children’s in-school and after school physical activities, transportation to/from schools and sedentary behaviors to obtain the total MET.

#### Other dietary intake variables

The data utilized the 2002 version of Food Composition Table (FCT) to calculate macronutrient intake values for the dietary data. Caloric, fat and carbohydrate intake were obtained from 3-day 24-hour recalls reported by children’s parents. We dichotomized caloric, fat and carbohydrate intake based on data distribution as well as dietary recommendations[20]. However, we did not include dietary intakes in our regression analyses due to their strong associations with FFC; instead, we looked into their relationships with FFC patterns.

Gender was treated as an effect modifier, and we stratified our analyses by gender.

In addition, we examined BMI z-score and FFC by gender, age, income (low, median, and high), geographical region (East China, Northeast, South China, Central China and Southwest), and urbanicity (urban, and rural). Northeast includes Liaoning and Heilongjiang provinces; East China includes Jiangsu and Shandong; Central China includes Henan, Hubei, and Hunan; South China includes Guangxi, and Southwest includes Guizhou.

### Statistical Analysis

First, we explored the FFC patterns among children in China in 2004 and 2009. We examined FFC patterns among children who participated in both waves in 2004 and 2009, and later looked at children of the same age cohort in both waves. We used McNemar tests and Chi-squared tests respectively, to examine if there were any significant differences in terms of the percentage of fast food consumers over time.

Next, we tested the associations between FFC and BMI z-score and weight status in children. Note that in our models, since the prevalence of obesity was low, we combined the prevalence of overweight and obesity. We conducted linear regression analysis to examine the association between BMI z-scores and FFC, and logistic regression analysis to examine the association between overweight/obesity with FFC. Statistical significance was set at *P*<0.05. Analysis was conducted using Stata (Version 11.1, StataCorp).

We fit a linear regression model to examine the association between FFC and BMI z-scores in boys and girls. The model was specified as:

$$y=\beta_{0}+\beta_{1}\;*\;FFC+\beta_{2}\;*\;\text{cov}+\varepsilon$$ (1)

Where BMI z-score is the dependent variable. We conducted stratified analysis by gender to estimate the association between FFC and BMI z-scores while adjusting for age, household income, urbanicity, region and physical activity.

Similarly, we fit logistic regression models with children’s weight status as outcome.

Finally, we tested the longitudinal associations between FFC and BMI z-score and weight status.

BMI z-score [or log(odds of being overweight or obese)] = covariates

$$y_{1}=\beta_{0}+\beta_{1}\;*\;{FFC}_{0}+\beta_{2}\;*\;\text{cov}+\beta_{3}\;*\;BMIz-{score}_{0}+\varepsilon_{k}$$ (2)

Where we estimated the effect of FFC at baseline on BMI z-score and log odds of being overweight or obese during follow-up, while adjusting for baseline BMI z-score, physical activity, age, household income, urbanicity, and region, and stratified our analyses by gender.

## Results

### Study Sample Characteristics

**Table 1**shows the subjects’ socio-demographic characteristics. On average, children in wave 2009 were slightly younger than those in 2004, had higher family income, with more residing in South China and fewer in the Northeast region, consumed fewer daily calories, had lower physical activity levels, and had higher average BMI z-scores. Other characteristics were comparable in 2004 and 2009.

**Table 1:** Socio-demographic characteristics of children aged 6–17 years in nine provinces in China: China Health and Nutrition Survey 2004–2009 (cross-sectional analysis).

	Year 2004 (n = 1542)	Year 2009 (n = 1114)	With data in both waves (n = 376)
Age (years), mean (SD)	12.38 (3.3)	11.56 (3.2)	9.03 (1.8)
Girls, n (%)	723 (46.9)	493 (44.3)	166 (44.2)
Han Ethnicity, n (%)	1,303 (84.8)	919 (83.1)	303 (80.1)
Income/capita(RMB, yuan)^†^^,^^‡^, mean (SD)			
Low	1,445.8 (800.3)	2,370.0 (1474.2)	1431.1 (776.6)
Medium	4,378.6 (942.7)	6,772.1 (1,426.1)	4526.9 (927.4)
High	11,556.8 (6,279.4)	19,112.2 (14,723.4)	11421.6 (4729.0)
Urban residents, *n* (%)	447 (29.0)	305 (27.4)	105 (27.9)
Region^§^, *n* (%)			
Northeast	350 (22.7)	166 (14.9)	81 (21.5)
East China	255 (16.5)	200 (18.0)	62 (16.5)
Central China	492 (31.9)	337 (30.3)	86 (22.9)
South China	220 (14.3)	212 (19.0)	72 (19.2)
Southwest	225 (14.6)	199 (17.9)	75 (20.0)
Caloric intake (kcal)^┃^, mean (SD)	1,909.5 (679.5)	1,715.6 (622.1)	1636.1 (590.2)
Fat intake (% kcal)^┃^, mean (SD)	28.8 (10.7)	29.2 (11.0)	28.9 (10.7)
Carbohydrate intake (% kcal)^┃^, mean (SD)	59.1 (10.9)	57.9 (11.1)	59.1 (11.0)
Physical activity level ^||^ (MET in kcal/(kg*h), mean (SD)	4,426.0 (4,651.7)	3,949.0 (3,410.4)	3666.1 (3377.7)
BMI z-score^¶^	-0.01 (0.9)	0.02 (1.1)	0.01 (1.0)

Note: SD, standard deviation. MET, metabolic equivalent of task. BMI, body mass index.

† Annual per capita household income inflated to year 2011 currency values.

‡ Income groups were categorized based on annual per capita household income tertiles in that wave. In year 2004, low income ranged from -1,206.0 to 2,797.2 yuan, medium income ranged from 2,803.7 to 6,190.3 yuan, high income ranged from 6,196.2 to 60,557.5 yuan. In year 2009, low income ranged from -10,347.3 to 4,483.4 yuan, medium income ranged from 4,501.2 to 9,562.9 yuan, high income ranged from 9,578.8 to 168,998.9 yuan.

§ Regions include: Northeast (Liaoning, Heilongjiang), East China (Jiangsu, Shandong), Central China (Henan, Hubei, Hunan), South China (Guangxi), Southwest China (Guizhou).

^┃^ Caloric, fat and carbohydrate intakes were obtained from 3-day 24-hour recalls.

|| MET was obtained by aggregating childrenre in-school and after school physical activities, transportations to/from schools and sedentary behaviors.

^¶^ Weight and height was measured by trained clinical staffs. BMI was calculated from weight (kg) divided by height (meter) squared. BMI z-scores were calculated based on children’s age and gender groups within this sample.

### Trends in Fast Food Consumption

**Table 2**presents the time trends in the proportion of fast food consumers by socio-demographics, lifestyles and weight status for the same cohort aged 6–12.99 years in 2004 (thus aged 11–17.99 years in 2009). The proportion of fast food consumers rose from 18.5% in 2004 to 23.9% in 2009 (*P*<0.001). The increase was greater in boys (16.3% to 21.1%, *P*<0.001) than in girls (23.6% to 24.4%, *P*<0.001). The figure in minority ethnic group almost tripled (*P*<0.001) over time, far faster than that in Han people.

**Table 2:** Longitudinal analysis: Trends in the percentage (%) of Western fast food consumers among Chinese school-age children followed up from 2004 to 2009 (the same cohort, n = 376), by socio-demographics, lifestyles and weight status: China Health and Nutrition Survey 2004–2009^†^.

	Year 2004	Year 2009	p^§^	Trend^¶¶^
	%^‡^	%^‡^		
All^┃^	18.5	23.9	.000	↑
Gender				
Boy	16.3	21.1	.000	↑
Girl	23.6	24.4	.000	↑
Ethnicity				
Han	21.0	26.0	.000	↑
Not Han (minorities)	6.0	15.9	.000	↑
Household income (per capita) ^||^				
Low	10.3	19.2	.000	↑
Medium	11.2	18.5	.000	↑
High	36.2	35.1	.002	↓
Residence				
Urban	38.0	43.3	.10	↑
Rural	11.0	15.9	.000	↑
Region^¶^				
Northeast	17.5	29.0	.000	↑
East China	45.2	44.3	.37	↓
Central China	20.7	19.8	.000	↓
South China	5.7	13.4	.000	↑
Southwest	5.8	15.7	.000	↑
Dietary intake				
Caloric intake (kcal)^††^				
<1,200	13.9	13.9	.000	↓
≥1,200	20.0	25.2	.000	↑
Fat intake (as % of total energy intake)^††^				
≤35	15.7	18.6	.000	↑
>35	26.6	41.0	.001	↑
Carbohydrate intake (as % of total energy intake)^††^				
≤50	34.3	47.8	.07	↑
>50	14.5	18.3	.000	↑
Physical activity level (MET in kcal/(kg*h))^‡‡^				
Less active ≤5,000	16.9	23.7	.000	↑
Active >5,000	26.2	24.7	.000	↓
Weight Status (based on BMI)^§§^				
Under/normal weight	17.5	24.3	.000	↑
Overweight	22.9	18.8	.18	↓
Obese	42.9	50.0	.000	↑

Note: MET, metabolic equivalent of task. Results came from a sample of children who participated in both waves 2004 and 2009. Imputation treated any missing responses or those answered “unknown” as “0” when there was no fast food restaurant in the respondent's community.

^†^ Age is not listed here as children in waves 2004 and 2009 belong to different age groups.

‡ % represents the % of consumers among that specific sample.

§P-value for McNemar tests to examine if there was any significant difference between the percentage of fast food consumers in wave 2004 and that in wave 2009.

| Questions on fast food consumption frequency: “During the past 3 months, how many times have you eaten at a Western fast food restaurant, such as McDonald’s or Kentucky Fried Chicken?” Responses to this question were dichotomized into 1: consumed at least once or 0: not consumed or unknown to calculate the percentage of fast food consumers.

|| Annual per capita household income inflated to year 2011yuan currency values.

¶ Regions include: Northeast (Liaoning, Heilongjiang), East China (Jiangsu, Shandong), Central China (Henan, Hubei, Hunan), South China (Guangxi), Southwest China (Guizhou).

^¶¶^ ↑: increase, from 2004 to 2009; ↓: decrease, from 2004 to 2009.

†† Caloric, fat and carbohydrate intake were obtained from 3-day 24-hour recalls. They were dichotomized based on data distribution as well as dietary recommendations.

‡‡ MET was aggregated from children’s in-school and after school physical activities, transportations to/from schools and sedentary behaviors.

§§ Weight and height was measured by trained clinical staffs. BMI was calculated as weight (kg) divided by height (meter) squared. Weight status was determined based on the International Obesity Task Force (IOTF) gender- and age-specific BMI cut-offs.

↑: increase; ↓: decrease; →: no change from 2004 to 2009.

The rise in the percentage of fast food consumers was most pronounced in the low- and median- income groups (10.3% to 19.2% in low-income households; 11.2% to 18.5% in medium- income households, both *P*<0.001). It decreased significantly in the high-income group (36.2% to 35.1%, *P*<00.01).

The same trends existed among the urban and rural residents. The percentage increased dramatically in both urban (38.0% to 43.3%, *P*<0.05) and rural residents (11.0% to 15.9%, *P*<0.001).

While the percentage consumed increased dramatically in the Northeast (17.5% to 29.0%, *P*<0.001), South (5.7% to 13.4%, *P*<0.001) and Southwest regions (5.8% to 15.7%, *P*<0.001), it decreased slightly in the remaining regions.

In terms of dietary intakes, the percentage of fast food consumers increased significantly (20% to 25.2%, *P*<0.001) among those who consumed 1,200 kcal or more calories per day. The percentage of fast food consumers increased in all fat and carbohydrate intake subgroups.

Compared to 2004, more children with a low physical activity level (MET in kcal/(kg*h) ≤5,000) consumed fast food in 2009 (16.9% in 2004 to 23.7% in 2009, *P*<0.001), while fewer children with a high physical activity level (MET in kcal/(kg*h) >5,000) consumed fast food (26.2% in 2004 to 24.7% in 2009, *P*<0.001). Among children with different weight status, more underweight or normal weight children consumed fast food in 2009 (19.7% in 2004 to 25.1% in 2009, *P*<0.001), compared to to those who were overweight (22.9% in 2004 to 18.8% in 2009, *P*<0.001),

**Table 3**shows the trends in the percentage of fast food consumers by socio-demographics, lifestyles and weight status characteristics for participants of the same-age in 2004 vs. those in 2009, i.e., to examine the time trends controlling for age. From 2004 to 2009, more adolescents aged 13–17 reported consuming fast food, while it did not change much in children aged 6–10. For children aged 13–17, there was a dramatic increase (17.9% to 26.3%, P<0.01). Among children aged 6–10, it did not change much, but it more than tripled among ethnic minorities (5.2% to 16.9%, *P*<0.05), while it decreased in East China (where the proportion was high in 2004) over time (43.3% to 32.1%, *P*<0.01).

**Table 3:** Time trends in the percentage (%) of Western fast food consumers among Chinese children of the same age between 2004 and 2009, by age group (those aged 6–10 vs 13–17 years old), socio-demographics, lifestyles and weight status: China Health and Nutrition Survey.

	Children aged 6–10 in 2004 or 2009 (N = 1534)				Children aged 13–17 in 2004 or 2009 (N = 362)			
	Year 2004	Year 2009	Sig.^†^	Trend^¶¶^	Year 2004	Year 2009	Sig.^†^	Trend^¶¶^
	%	%			%	%		
Among all^§^	18.1	18.3		→	**17.9**	**26.3**	***	↑
Age (years)^┃^								
6–8 or 13–15	18.2	17.1		→	16.2	24.3		↑
9–10 or 16–17	18.1	19.9		→	21.3	31.7		↑
Gender								
Boys	15.3	18.7		→	**15.0**	**25.0**	**	↑
Girls	21.2	17.7		→	21.3	27.8		↑
Ethnicity								
Han	21.1	18.5		→	**19.1**	**27.6**	**	↑
Not Han (minorities)	**5.2**	**16.9**	*	↑	9.5	19.0		↑
Household income/capita (yuan) ^||^								
Low	7	9.2		→	13.3	17.7		↑
Medium	9.9	14.8		→	**13.7**	**22.2**	*	↑
High	39.3	32.0		→	26.9	36.2		↑
Urbanicity								
Urban	42.1	42.3		→	34.3	38.8		→
Rural	9.7	9.8		→	**10.3**	**20.6**	***	↑
Region^¶^								
Northeast	23.6	28.1		→	19.3	29.7		↑
East China	**43.3**	**34.1**	**	↓	**23.4**	**41.2**	**	↑
Central China	13.4	15.4		→	15.7	24.2		↑
South China	6.8	6.0		→	15.4	15.2		→
Southwest	5.3	13.3		→	15.0	21.2		↑
Weight Status^§§^								
Under/normal weight	16.5	17.5		→	**17.4**	**26.2**	***	↑
Overweight	27.3	26.7		→	30.3	26.5		→
Obese	33.3	16.7		→	0.0	33.3		↑
Dietary intakes								
Caloric intake (kcal)^††^								
<1,200	14.9	12.3		→	22.9	21.2		→
≥1,200	19.6	20.1		→	**18.3**	**26.7**	**	↑
Fat intake (as % of total energy intake)^††^								
≤35	14.7	14.4		→	16.8	22.4		↑
>35	28.5	25.9		→	**23.2**	**36.8**	*	↑
Carbohydrate intake (as % of total energy intake)^††^								
≤50	36.1	25.6		→	**26.2**	**41.3**	*	↑
>50	13.6	15.3		→	**16.6**	**22.2**	*	↑
Physical activity level (MET in kcal/(kg*h))^‡‡^								
≤5,000	16.6	16.8	→	**16.5**	**24.4**	*	*	↑
>5,000	26.6	24.2	→	**20.7**	**30.8**	*	*	↑

Note: MET, metabolic equivalent of task. Results came from two samples of children who aged 6–10.99 years in waves 2004 and of the same age in 2009, or children who aged 13–17.99 years in waves 2004 and of the same age in 2009. Imputation treated any missing responses or those answered “unknown” as “0” when there was no fast food restaurant in the respondent's community.

| Age group 6–8.99 for children aged 6–10.99, and age group 13–15.99 for children aged 13–17.99. Age group 9–10.99 for children aged 6–10.99, and age group 16–17.99 for children aged 13–17.99. The age range of 6–10.99 and 13–17.99 were chosen so that there were e no overlaps between children of the same age range in wave 2004 and 2009.

^†^ * *P* < .05; ** *P* < .01; *** *P* < .001 for Chi-squared tests to examine if there was any significant difference between the percentage of fast food consumers in wave 2004 and that in wave 2009.

^§^ Questions on fast food consumption frequency: “During the past 3 months, how many times have you eaten at a Western fast food restaurant, such as McDonald’s or Kentucky Fried Chicken?” Responses to this question were dichotomized into 1: consumed at least once or 0: not consumed or unknown to calculate the percentage of fast food consumers.

|| Annual per capita household income inflated to year 2011 yuan currency values.

^¶^ Regions include: Northeast (Liaoning, Heilongjiang), East China (Jiangsu, Shandong), Central China (Henan, Hubei, Hunan), South China (Guangxi), Southwest China (Guizhou).

^††^ Caloric, fat and carbohydrate intake were obtained from 3-day 24-hour recalls. They were dichotomized based on data distribution as well as dietary recommendations.

^‡‡^ MET was aggregated from children’s in-school and after school physical activities, transportations to/from schools and sedentary behaviors.

^§§^ Weight and height was measured by trained clinical staffs. BMI was calculated from weight (kg) divided by height (meter) squared. Weight status was determined based on the International Obesity Task Force (IOTF)’s gender- and age-specific BMI cut-offs.

^¶¶^ ↑: increase; ↓: decrease; →: no change from 2004 to 2009.

FFC rates were significantly higher in the medium-income group (13.7% to 22.2%, *P*<0.05), among rural residents (10.3% to 20.6%, *P*<0.001), among those who consumed 12,00 kcal or more per day (18.3% to 26.7%, *P*<0.01), among those who consumed more fat (23.2% to 36.8%, *P*<0.05), and among those who were under or normal weight (17.4% to 26.2%, *P*<0.001). The consumption rate was higher in ball carbohydrate intake and physical activity level subgroups. The consumption rate was mostly stable in other groups.

### Association between Fast Food Consumption and Children’s Weight Status

Using linear and logistic regression analyses, we assessed cross-sectional associations between Chinese children’s FFC and BMI z-scores and weight status in both wave 2004 and 2009, respectively, with stratification by gender (**Table 4**). After controlling for potential confounders including age, ethnicity, household income, urbanicity, geographical region and physical activity levels, FFC was positively associated with BMI z-score (β = 0.13, 95% CI: -0.16, 0.42, β = 0.41, 95% CI: 0.04, 0.78 for boys in 2004 and 2009 respectively) and weight status (OR = 1.62, 95% CI: 0.52, 5.12, OR = 2.79, 95% CI: 0.87, 8.97 for boys in 2004 and 2009 respectively) in wave 2004, while it was not significant in 2009. However, most of the associations were not significant, except for that of FFC and BMI z-score among girls.

**Table 4:** Linear and logistic regression analysis for cross-sectional and longitudinal associations between Chinese children’s Western fast food consumption and their BMI z-scores and weight status: China Health and Nutrition Survey 2004–2009.

Consumed fast food ^|^ vs. not (ref.)	BMI z-score^†^^,^^‡^		Overweight or Obese^§^^,^ ^||^	
	β	(95% CI)	OR	(95% CI)
***Cross-sectional analysis***				
For wave = 2004				
Boys (n = 794)	0.13	(-0.16, 0.42)	1.62	(0.52, 5.12)
Girls (n = 703)	0.00	(-0.40, 0.40)	0.92	(0.26, 3.27)
For wave = 2009				
Boys (n = 612)	**0.41**	**(0.04, 0.78)**	2.79	(0.87, 8.97)
Girls (n = 480)	0.09	(-0.31, 0.49)	0.94	(0.25, 3.52)
***Longitudinal analysis***				
Boys (n = 210)	0.02	(-0.71, 0.75)	0.71	(0.38, 1.32)
Girls (n = 166)	-0.14	(-1.03, 0.75)	NA^J^	NA^J^

Note: OR = Odds Ratio, CI = Confidence Interval. NA, not applicable.

^┃^Questions on fast food consumption frequency: “During the past 3 months, how many times have you eaten at a Western fast food restaurant, such as McDonald’s or Kentucky Fried Chicken?” Responses to this question were dichotomized into 1: consumed at least once or 0: not consumed or unknown to calculate the percentage of fast food consumers.

^†^ Weight and height was measured by trained clinical staffs. BMI was calculated from weight (kg) divided by height (meter) squared. BMI z-scores were calculated based on children’s age and gender groups within this sample.

^‡^ Linear regression models: For cross-sectional data analyses, BMI z-scores in year 2004/2009 regressed on fast food consumption in the same year stratified by gender, after controlling for age, ethnicity, household income, urbanicity, geographical region and physical activity levels. For longitudinal data analyses, BMI z-scores in year 2009 regressed on baseline fast food consumption stratified by gender, after controlling for baseline age, ethnicity, household income, urbanicity, geographical region and physical activity levels.

^§^ Weight and height was measured by trained clinical staffs. BMI was calculated as weight (kg) divided by height (meter) squared. Weight status was determined based on the International Obesity Task Force (IOTF) gender- and age-specific BMI cut-offs.

^**||**^ Logistic regression models: For cross-sectional data analyses, the log odds of being overweight or obese in year 2004/2009 regressed on fast food consumption in the same year stratified by gender, after controlling for age, ethnicity, household income, urbanicity, geographical region and physical activity levels. For longitudinal data analyses, the log odds of being overweight or obese in year 2009 or not regressed on baseline fast food consumption stratified by gender, after controlling for baseline age, ethnicity, household income, urbanicity, geographical region and physical activity levels.

J Estimates could not be obtained as only 7 out of 161 girls were overweight or obese in 2009 in the sample.

Further linear and logistic regression analysis (**Table 4**) examined the longitudinal associations between FFC and BMI z-score and weight status, and stratified by gender. Our estimates suggest that FFC was associated with 0.02 (95% CI: -0.71, 0.75) higher BMI-z in boys, but 0.14 (95%CI: -1.03, 0.75) lower BMI-z in girls. However, none was statistically significant (p>0.05). Sensitivity analyses were conducted before FFC was imputed, and showed similar results (not shown here).

## Discussion

Our analysis of the nationwide survey data shows that FFC (excluding Chinese fast food) increased significantly among Chinese school-age children during 2004–2009, and the increase was especially rapid among some groups such as older children, boys, those from low- and medium-income families, and those from rural areas and East China, compared to their counterparts. During this period, FFC decreased in children who were from high-income families and those who were overweight. In almost all socio-demographic subgroups, as children aged, more respondents reported having consumed fast food in the past three months. This finding was likely to be fueled by the increased accessibility of Western fast food restaurants and children’s increased pocket money and independence. The trend was different by age group. FFC rate in children was relatively stable, while it increased rapidly in adolescents, from 17.9% to 26.3%. For adolescents, the increasing trend was prominent in boys, of Han ethnicity, from medium-income families, of rural residents, from East China, of under/normal weight, or those with a diet high in calories or fat. These findings are likely due to their increased independence and peer influence.

The Western fast food industry has been growing rapidly in China. Compared to a decade ago, nowadays children have better access to fast food and the food has become more affordable. Moreover, FFC is viewed favorably among children. A cross-cultural study on brand identity reported that Chinese children had more favorable impressions of KFC than did their US counterparts [21]. Despite these changes in the food environment in China, our findings indicate some large spatial-temporal disparities and time trends in FFC in adolescents between urban and rural regions: urban adolescents had more FFC. However, during 2004–2009, FFC increased in rural areas, but decreased in urban areas. Such spatial-temporal differential patterns mirror the inequalities of regional economic development and reflect the nonlinear effect of income rise on food-away-from-home (FAFH) consumption. It is known that an increase in income generates a greater increase in FAFH expenditure in high-income households than in low-income households in China[22]. On one hand, as an important component of FAFH, FFC increased dramatically in rural areas with an increase in rural household income. On the other hand, the effect of increasing household income in urban areas on FAFH was more pronounced for other foods. In high-income urban households, more awareness of health, media influence, and increased demand for high quality food in recent years may have resulted in a decrease in FFC, as FFC may be increasingly viewed as unhealthy for children. We have not observed such pattern in rural areas yet, likely because of nonlinear income effects at different threshold levels. Further research is warranted to explain what we observed.

By separating the age and period effects, we found different patterns. In general, as age increased, more children had consumed Western fast food, while the consumption prevalence in young children did not change much. This finding is likely because adolescents who had entered middle and high schools had more autonomy and pocket money, and were more likely to be under peer influence than younger kids [23,24]. Therefore, older children had more freedom and opportunities to consume Western fast food.

Interestingly, the FFC rate decreased in the children from high-income families, but those from low- and medium-income families increased their FFC. This result is consistent with the shifted nutrition transition and findings that the burden of obesity and metabolic risk has started to move from the better-off to the poorer population groups in some developing countries [25].

Although FFC generally increased, western FFC did not appear to be a large contributor of childhood obesity in China. We did not find a meaningful association between western FFC and child weight status using the CHNS data. Another study reported that less than 1% of Chinese children’s total calorie intake was obtained from fast food in 2000, while the proportion was 14.8% for children in the United States [26,27]. Other factors have contributed to childhood obesity in China, such as increased family income and increased consumption of animal-source food [28]. Also, using the CHNS data, Du et al. found that the density of fast food restaurants in neighborhood was not associated with BMI in men in rural China, while an increase of one fast food restaurant was associated with lower BMI (by 0.02) in rural women [29]. The CHNS data also show that overweight and obesity was not associated with fast food preference in adults, and food environment might play a smaller role than nutritional knowledge in influencing consumers’ consumption choices [30]. These findings for adults may indicate the complex relationship between FFC and weight outcomes in Chinese children as well.

Some empirical and review studies have examined the associations between FFC, increased fat and calorie intakes and childhood obesity[12,31]. However, most of these findings were based on studies conducted in high-income countries such as the United States. To our knowledge, thus far, Rosenheck’s (2008) review is the most comprehensive to date in its review of 16 studies (6 cross sectional-, 7 prospective cohort-, 3 experimental studies). It concluded that the association between FFC and weight gain was not clear, but sufficient evidence exists for public health recommendations to limit FFC and facilitate healthier menu selection. Of the 7 prospective cohort studies, 4 were conducted among children and adolescents, 6 found a positive association between more frequent FFC and an increase in total caloric intake or BMI. All 3 studies conducted among young adults reported an association between FFC and increased BMI. Period effect is a potential cause for our mixed findings. We found that the percentage of fast food consumers decreased from 2004 to 2009 among children from high- income families, but it increased in both low- and medium- income families. Over time with increased family income, more and more Chinese families can afford eating fast food on a regular basis. More future research is needed to study the impact of FFC on public health in China.

Our study helps fill two main gaps in the literature: only a small number of studies have tested the association between FFC and overweight/obese in developing countries, and very few previous studies have used longitudinal data. Two large cross-sectional studies have examined the association in China, but provided conflicting findings. Our previous cross-sectional study among 21,198 children in Beijing reported a positive association between FFC and overweight/obese[5], but another study among 9,023 adolescents from seven large cities in China reported a negative association[32].

Consistent with findings from a recent US study (29), our present study did not detect a significant association between FFC and childhood obesity. The lack of an association could be because FFC only accounts for a small proportion of Chinese children’s total energy intake, our sample size is relatively small, and there is measurement error in assessing FFC. One of our recent studies shows that the density of neighborhood restaurants was associated with BMI in rural Chinese adults[29]. Nevertheless, FFC may affect overweight/obesity indirectly. For example, FFC may have promoted western food culture among Chinese children, which may influence their other food intake and cause a shift to high energy density food and drinks at home or away from home.

The main strengths of our study include longitudinal analysis and stratified analysis to examine the time trends in FFC and the association between FFC and obesity in Chinese children. The study also has several limitations. First, only 376 children had complete follow-up data. The low follow-up rate was mainly because children were not at home during the follow-up data collection and some families had moved out of the previous community. Second, the definition of fast food was not clearly specified in the survey questionnaire. The survey question asked about the number of times children visited fast food restaurants like McDonald’s. In this case, other Western fast food restaurants may have not been taken into account.

In conclusion, more Chinese children have consumed Western fast food over time. The increase is dramatic and is more rapid in some groups such as among adolescents (vs. younger children), boys, of non-Han ethnicity, those from low- and medium-income families, and those from rural areas or East China. FFC has been linked with adverse dietary quality and health conditions. Efforts are needed to study the impact of FFC on health outcomes, as well as the methods to promote healthy eating among young people in China.

## References

[1] de Onis M, Blossner M, Borghi E (2010) Global prevalence and trends of overweight and obesity among preschool children. Am J Clin Nutr 92: 1257–1264. 10.3945/ajcn.2010.29786 20861173

[2] Wang Y, Lobstein T (2006) Worldwide trends in childhood overweight and obesity. Int J Pediatr Obes 1: 11–25. 17902211 10.1080/17477160600586747

[3] Wang Y, Mi J, Shan XY, Wang QJ, Ge KY (2007) Is China facing an obesity epidemic and the consequences? The trends in obesity and chronic disease in China. Int J Obes (Lond) 31: 177–188. 16652128 10.1038/sj.ijo.0803354

[4] Ogden CL, Carroll MD, Kit BK, Flegal KM (2012) Prevalence of obesity and trends in body mass index among US children and adolescents, 1999–2010. JAMA 307: 483–490. 10.1001/jama.2012.40 22253364 PMC6362452

[5] Shan XY, Xi B, Cheng H, Hou DQ, Wang Y, et al (2010) Prevalence and behavioral risk factors of overweight and obesity among children aged 2–18 in Beijing, China. Int J Pediatr Obes 5: 383–389. 10.3109/17477160903572001 20233154

[6] Wang Y, Wu Y, Wilson RF, Bleich S, Cheskin L, et al. (2013) Childhood obesity prevention programs: comparative effectiveness review and meta-analysis. 23865092

[7] Pingali P (2007) Westernization of Asian diets and the transformation of food systems: implications for research and policy. Food policy 32: 281–298.

[8] Popkin BM (1999) Urbanization, lifestyle changes and the nutrition transition. World Development 27: 1905–1916.

[9] KFC (2012) KFC in China.

[10] McDonald’s (2014) Restaurant List.

[11] Vertinsky I (2002) Advertising trends in urban China. Journal of Advertising Research 42: 73–81.

[12] Rosenheck R (2008) Fast food consumption and increased caloric intake: a systematic review of a trajectory towards weight gain and obesity risk. Obes Rev 9: 535–547. 10.1111/j.1467-789X.2008.00477.x 18346099

[13] Duffey KJ, Gordon-Larsen P, Jacobs DR Jr., Williams OD, Popkin BM (2007) Differential associations of fast food and restaurant food consumption with 3-y change in body mass index: the Coronary Artery Risk Development in Young Adults Study. Am J Clin Nutr 85: 201–208. 17209197 10.1093/ajcn/85.1.201

[14] Pereira MA, Kartashov AI, Ebbeling CB, Van Horn L, Slattery ML, et al (2005) Fast-food habits, weight gain, and insulin resistance (the CARDIA study): 15-year prospective analysis. Lancet 365: 36–42. 15639678 10.1016/S0140-6736(04)17663-0

[15] Kamada T (1992) System biomedicine: a new paradigm in biomedical engineering. Frontiers of medical and biological engineering: the international journal of the Japan Society of Medical Electronics and Biological Engineering 4: 1. 1599879

[16] Zhang B, Zhai FY, Du SF, Popkin BM (2014) The China Health and Nutrition Survey, 1989–2011. Obes Rev 15 Suppl 1: 2–7. 10.1111/obr.12119 PMC3869031 24341753

[17] Ng SW, Norton EC, Guilkey DK, Popkin BM (2012) Estimation of a dynamic model of weight. Empirical Economics 42: 413–443.

[18] Cole TJ, Bellizzi MC, Flegal KM, Dietz WH (2000) Establishing a standard definition for child overweight and obesity worldwide: international survey. BMJ 320: 1240–1243. 10797032 10.1136/bmj.320.7244.1240 PMC27365

[19] Ainsworth BE, Haskell WL, Whitt MC, Irwin ML, Swartz AM, et al (2000) Compendium of physical activities: an update of activity codes and MET intensities. Med Sci Sports Exerc 32: S498–504. 10993420 10.1097/00005768-200009001-00009

[20] Macronutrients IoMPo, Intakes IoMSCotSEoDR (2005) Dietary reference intakes for energy, carbohydrate, fiber, fat, fatty acids, cholesterol, protein, and amino acids: Natl Academy Pr. 10.1016/s0002-8223(02)90346-9 12449285

[21] Witkowski TH, Ma Y, Zheng D (2003) Cross-cultural influences on brand identity impressions: KFC in China and the United States. Asia Pacific Journal of Marketing and Logistics 15: 74–88.

[22] Ma H, Huang J, Fuller F, Rozelle S (2006) Getting rich and eating out: consumption of food away from home in urban China. Canadian Journal of Agricultural Economics/Revue canadienne d'agroeconomie 54: 101–119.

[23] Houldcroft L, Haycraft E, Farrow C (2014) Peer and friend influences on children's eating. Social Development 23: 19–40.

[24] Verstraeten R, Van Royen K, Ochoa-Aviles A, Penafiel D, Holdsworth M, et al (2014) A conceptual framework for healthy eating behavior in ecuadorian adolescents: a qualitative study. PLoS One 9: e87183 10.1371/journal.pone.0087183 24489865 PMC3906122

[25] Popkin BM (2014) Synthesis and implications: China's nutrition transition in the context of changes across other low- and middle-income countries. Obes Rev 15 Suppl 1: 60–67. 10.1111/obr.12120 24341759 PMC3869101

[26] Adair LS, Popkin BM (2005) Are child eating patterns being transformed globally? Obesity research 13: 1281–1299. 16077000 10.1038/oby.2005.153

[27] Popkin BM (2008) Will China’s nutrition transition overwhelm its health care system and slow economic growth? Health Affairs 27: 1064–1076. 10.1377/hlthaff.27.4.1064 18607042 PMC2447919

[28] Zhai F, Wang H, Du PhD S, He Y, Wang Z, et al (2007) Lifespan nutrition and changing socio-economic conditions in China. Asia Pacific journal of clinical nutrition 16: 374 17392135

[29] Du W, Su C, Wang H, Wang Z, Wang Y, et al (2014) Is density of neighbourhood restaurants associated with BMI in rural Chinese adults? A longitudinal study from the China Health and Nutrition Survey. BMJ open 4: e004528 10.1136/bmjopen-2013-004528 24755211 PMC4010850

[30] Zhang X, van der Lans I, Dagevos H (2012) Impacts of fast food and the food retail environment on overweight and obesity in China: a multilevel latent class cluster approach. Public health nutrition 15: 88–96. 10.1017/S1368980011002047 21896233

[31] Fraser LK, Clarke GP, Cade JE, Edwards KL (2012) Fast food and obesity: a spatial analysis in a large United Kingdom population of children aged 13–15. Am J Prev Med 42: e77–85. 10.1016/j.amepre.2012.02.007 22516506

[32] Hsu Y-W, Johnson CA, Chou C-P, Unger JB, Sun P, et al (2011) Correlates of overweight status in Chinese youth: an East-West paradox. American journal of health behavior 35: 496–506. 22040595 10.5993/ajhb.35.4.11

